# First illustration of chromosomes and genetic system of Lecanodiaspidinae (Homoptera, Coccinea, Asterolecaniidae s.l.)

**DOI:** 10.3897/CompCytogen.v12i3.29648

**Published:** 2018-10-04

**Authors:** Ilya A. Gavrilov-Zimin

**Affiliations:** 1 Zoological Institute, Russian Academy of Sciences, Universitetskaya nab. 1, St. Petersburg, 199034, Russia Zoological Institute, Russian Academy of Sciences St. Petersburg Russia

**Keywords:** *
Psoraleococcus multipori
*, scale insects, morphology, karyotype, Lecanoid system

## Abstract

The karyotype of *Psoraleococcusmultipori* (Morrison, 1921) was studied for the first time, based on material from Indonesia (Sulawesi). The diploid chromosome number was found to be 18 in both males and females, but some cells contained also additional small chromosomal elements, probably B chromosomes. About 50 % of the studied embryos demonstrated paternal genome heterochromatinization of one haploid set of chromosomes (PGH) suggesting presence of a Lecanoid genetic system. The embryos with PGH are known to be always the male embryos in scale insects and so, bisexual reproduction may be presumed for *P.multipori*. The information provided represents the first probative cytogenetic data for the subfamily Lecanodiaspidinae Targioni Tozzetti, 1896 as a whole. A detailed morphological figure and photos of female and male embryonic cells are given. Additionally, it was discovered that the females of *P.multipori* exhibit complete ovoviviparity.

The subfamily Lecanodiaspidinae Targioni Tozzetti, 1896 comprises 12 genera and about 80 species in the world fauna (Ben-Dov 2006). Most species are delicate insects with vestigial or partly reduced legs in adult females, covered by a resinous translucent or semi-translucent protective test. All species are obligate phytophages on angiosperm plants, most frequently on trees and shrubs. The subfamily is often considered as a separate family (see, for example, Koteja 1974, Ben-Dov 2006, Hodgson and Williams 2016 and others). However, Lecanodiaspidinae along with Asterolecaniinae Cockerell, 1896 and Cerococcinae Balachowsky, 1942 share a well-defined apomorphic character – the presence of so-called 8-shaped pores – the peculiar wax glands scattered on dorsum and/or venter of adult females and larvae (Fig. 1). Due to this character all three groups are traditionally considered as subfamilies of Asterolecaniidae s.l. (see, for example, Brown and McKenzie 1962, Danzig 1980, Danzig and Gavrilov-Zimin 2014, and Gavrilov-Zimin 2018 and references therein).

Lecanodiaspidinae was almost unstudied previously in respect of cytogenetics. The chromosomal number (2n=14) was reported for one species only, *Anomalococcusindicus* Ramakrishna Ayyar, 1919 by Parida and Moharana (1982) without karyotype photo or information about genetic system. Two other subfamilies of Asterolecaniidae are also very poorly studied cytogenetically with only one analyzed species for subfam. Asterolecaniinae (Gavrilov 2007) and 3 species for subfam. Cerococcinae (Brown 1959, Brown and McKenzie 1962); the chromosome numbers of these species vary from 2n=6 to 2n=24 (l.c.).

**Figure 1.**
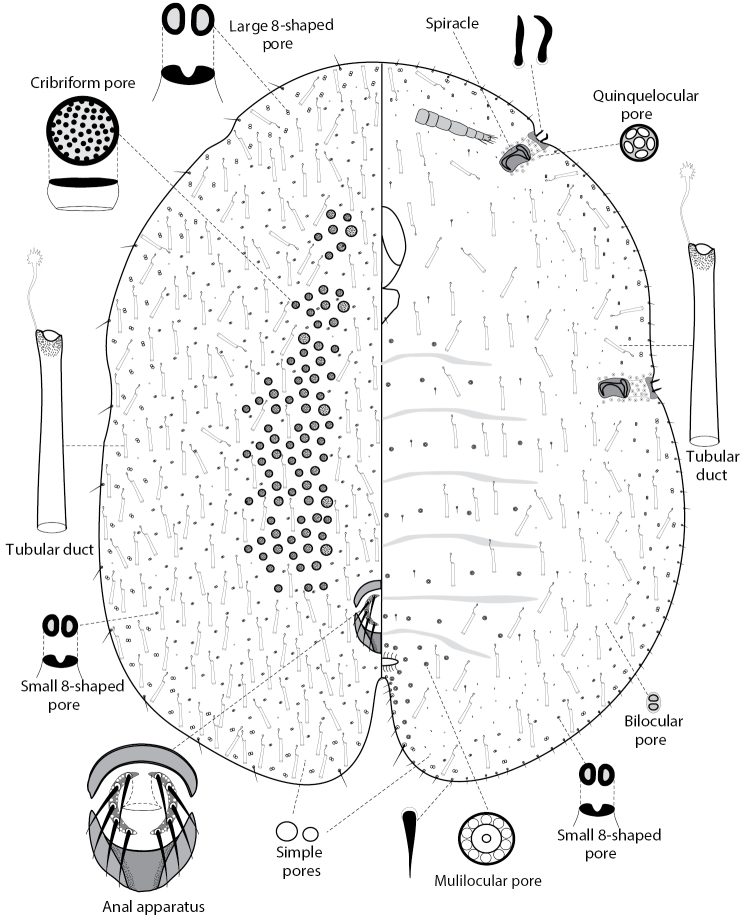
Morphology of *Psoraleococcusmultipori*, adult female, Indonesia (Sulawesi).

During an expedition in Sulawesi Is. (Indonesia) the author was able to collect the series of adult females of *Psoraleococcusmultipori* (Morrison, 1921): K 923, vicinity of Kendari, on branch of undetermined dicotyledonous tree, inside of ant gallery, 10.XI.2011, I. Gavrilov-Zimin (deposited at Zoological Institute RAS, St. Petersburg). These females appeared to be suitable for preparing both morphological and chromosomal slides. The method of preparation of the morphological slides and method of squashing of the embryonic cells in lactoacetorcein for chromosomal studies see, for example, in Gavrilov-Zimin (2018).

The diploid chromosomal number of *P.multipori* is 18 in both sexes (Figs 2a, f). The karyotype consists of chromosomes gradually differing in size (Fig. 2f). A similar gradual pattern of chromosome size variation (for karyotype 2n=14) was reported for *Anomalococcusindicus* by Parida & Moharana (1982). Some cells of *P.multipori* contain additional small chromosomal elements (Fig. 2b), probably B-chromosomes, which are also known in some scale insects from different families (see for review Gavrilov 2007), but were not reported previously for any Asterolecaniidae s.l. Totally, nine cleavage stage embryos were found in the two dissected females (in addition to numerous embryos at later stages) and five of them demonstrated characteristic Lecanoid heterochromatinization of one haploid set of chromosomes (Fig. 2c–d) that suggests a Lecanoid genetic system and is known for many other neococcids (superfamily Coccoidea) (Nur 1980, Gavrilov 2007, Gavrilov-Zimin 2016). In particular, within Asterolecaniidae s.l., such a system (including both “Lecanoid” and “Comstockioid” variants of spermatogenesis) was demonstrated previously by Brown (1959) for *Cerococcusquercus* Comstock, 1882 and for *Mycetococcusehrhorni* (Cockerell, 1895) by Brown and McKenzie (1962) (both species are from the subfamily Cerococcinae). The embryos with such heterochromatinization are always the males and so, bisexual reproduction may be presumed in *Psoraleococcusmultipori*. On the other hand, the adult males or male larvae have not been collected up to now in any species of the genus *Psoraleococcus* Borchsenius, 1960. This situation is probably connected with separate lives of female and male colonies on different parts of a host plant or even on different plants. Moreover, all species of *Psoraleococcus* live in symbiosis with ants (Lambdin and Kosztarab 1973) which may transport different instars of scale insects inside hidden underground galleries, which significantly impedes their detection and collection.

The females of *P.multipori* exhibit complete ovoviviparity, i.e. all stages of embryonic development occur inside the maternal body (see a review of reproductive strategies of scale insects and appropriate terminology in Gavrilov-Zimin 2018).

The work was performed in the frame of the state research project no. AAAA-A17-117030310018-5 at Zoological Institute, Russian Academy of Sciences.

**Figure 2.**
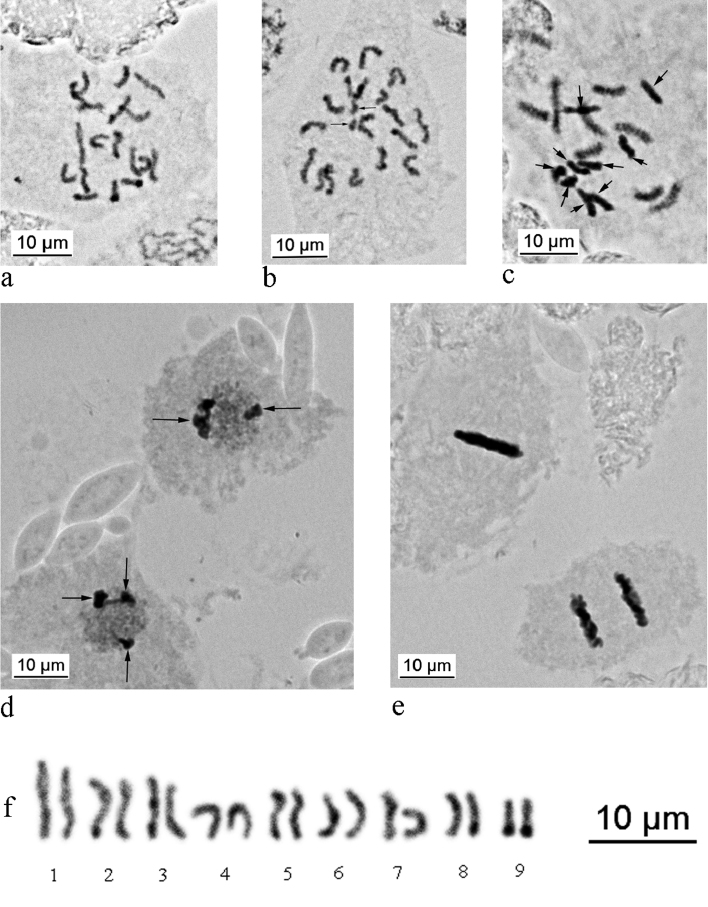
Embryonic cells of *Psoraleococcusmultipori*. **a** female embryo, 2n = 18 **b** female embryo, 2n = 18 + 2B (B-chromosomes arrowed) **c** male embryo, paternal set of chromosomes begins heterochromatinization (arrowed) **d** Lecanoid heterochromatinization (arrowed) in interphase cells of male embryo; **e** metaphase and anaphase in female embryo showing no lagging chromosomes (Bs) **f** karyogram, prepared basing on the Fig. 2a.
